# A gene pattern mining algorithm using interchangeable gene sets for prokaryotes

**DOI:** 10.1186/1471-2105-9-124

**Published:** 2008-02-26

**Authors:** Meng Hu, Kwangmin Choi, Wei Su, Sun Kim, Jiong Yang

**Affiliations:** 1 EECS, Case Western Reserve University, Cleveland, OH 44106 USA; 2 School of Informatics, Indiana University, Center for Genomics and Bioinformatics, Indiana University, Bloomington, IN 47408, USA

## Abstract

**Background:**

Mining gene patterns that are common to multiple genomes is an important biological problem, which can lead us to novel biological insights. When family classification of genes is available, this problem is similar to the pattern mining problem in the data mining community. However, when family classification information is not available, mining gene patterns is a challenging problem. There are several well developed algorithms for predicting gene patterns in a pair of genomes, such as FISH and DAGchainer. These algorithms use the optimization problem formulation which is solved using the dynamic programming technique. Unfortunately, extending these algorithms to multiple genome cases is not trivial due to the rapid increase in time and space complexity.

**Results:**

In this paper, we propose a novel algorithm for mining gene patterns in more than two prokaryote genomes using interchangeable sets. The basic idea is to extend the pattern mining technique from the data mining community to handle the situation where family classification information is not available using interchangeable sets. In an experiment with four newly sequenced genomes (where the gene annotation is unavailable), we show that the gene pattern can capture important biological information. To examine the effectiveness of gene patterns further, we propose an ortholog prediction method based on our gene pattern mining algorithm and compare our method to the bi-directional best hit (BBH) technique in terms of COG orthologous gene classification information. The experiment show that our algorithm achieves a 3% increase in recall compared to BBH without sacrificing the precision of ortholog detection.

**Conclusion:**

The discovered gene patterns can be used for the detecting of ortholog and genes that collaborate for a common biological function.

## Background

As more genome sequences become available, comparing multiple genomes becomes an important research method that can lead to the discovery of new biological insights. In this paper, we are interested in discovering sets of grouped protein genes (hereafter referred to as genes), that are present in unknown subsets of genomes. In general, we do not have prior knowledge in which sets of genes are commonly present in which sets of genomes, thus this is a data mining problem.

In [1] and [2], the authors presented a method to detect conserved clusters of genes, called PCBBH (Pair of Close Bidirectional Best Hits), which are pairs of genes that appear close to each other in multiple genomes. However, the problem of this approach is that it only identifies pairs of genes that are conserved clustered in genomes. The number of discovered pairs is too large for interpreting. On the other hand, there are several well developed algorithms for predicting gene patterns in a pair of genomes. Indeed, this problem is similar to the well known biological sequence alignment problem. There are successful local multiple sequence alignment (motif) algorithms, such as Pratt [3,4] and TEIRESIAS [5], based on a character enumeration approach. We argue that the pattern mining problem can be solved by the enumeration approach, as opposed to the optimization approach, since the basic unit to enumerate is a gene or family, not a single character.

In the case where family classification information of genes in the genome set is known, this problem can be thought as predicting sets of families in multiple genomes by interpreting a genome as a sequence of families. This requires well designed algorithms for several reasons. First, two predicted family sequences in two different genomes, say *G*_*A *_and *G*_*B*_, can be different since the order of families can be different and some families can appear multiple times. Second, we do not know which genomes, *G*_*A *_and *G*_*B *_in this case, share a common family sequence. Prediction of family sequences leverages the fact that functionally related genes occur in a physically clustered form, especially operons in bacteria. This observation leads to an interesting problem formulation known as the *gene team model *[6,7], which searches for a set of gene groups that co-occur in a pair of closely related genomes. In particular, these algorithms use COG families [8,9] for a *pair *of genomes [6,7] and the problem is referred to as *COG teams*. [9] presents an improvement of the COGNITOR program that is used to fit new proteins into the COGs. The original COGNITOR program uses multiple genome-specific best hits (BeTs) as the only criterion for assigning new proteins to COGs. [9] introduced an estimate of the probability that the query protein is assigned to the given COG by chance. Under the assumption of uniform distribution, they calculate the probability of a specified number of BeTs into a particular COG. When the specified number of BeTs is large, they calculate the probability in a simplified way. The requirement of all genes being within a certain physical distance, say 300 bp, is often too strict. Recently, we extended the gene team model by defining and using the hybrid gene team model [10,11]. We will call the problem of predicting family sequences common to multiple genomes the *family pattern mining problem*.

However, there are many genomes whose genes have not been classified into families. For example, only about 100 genomes are well characterized as COG teams among more than 300 genomes available at GenBank. This requires putative family assignment of genes as well as gene patterns prediction.

Unfortunately family assignment of genes is still an open research problem. Thus this problem is more challenging than the family pattern mining problem, and this problem will be called the *gene pattern mining problem*. One of the most well accepted formulations of the gene pattern mining problem is to search for patterns with optimal scores. FISH [12] and DAGchainer [13] are two recent algorithms based on the optimization problem formulation. For example, FISH finds all maximal k-clumps for a directed acyclic graph where vertices are genes. If two genes are within a neighborhood distance, then there is an edge between the corresponding vertices. Since all gene matches are treated equally, the score of a clump is simply the number of points (gene matches), thus this problem can be easily formulated as a recursive scoring function, which can be solved using a dynamic programming technique. These two methods are primarily developed for a pair of genomes. The major hurdle to extending them for predicting patterns in multiple genomes is the computational complexity; the time and space complexity of the dynamic programming technique grow rapidly in regard to the number of genomes.

The main problem this paper solves is efficiently discovering gene patterns in multiple genomes where gene family classification is incomplete. The proposed algorithm can quickly identify gene patterns using the interchangeable gene sets from a large number of genome sequences. These genes patterns can be used to predict the annotation (or ortholog) of unknown genes. To validate the algorithm, we proposed the ortholog prediction method based on the gene pattern mining algorithm. This ortholog prediction outperforms BBH in ortholog identification which shows the usefulness of the gene patterns discovered by our algorithm. Our algorithm is necessary for two reasons: First, although there are methods to classify genes into families, there are plenty of cases where these gene classifications are incomplete, and discovering gene patterns in these cases remains an unsolved problem. Our proposed algorithm addresses this gene pattern mining problem by using the interchangeable gene sets. On the other hand, there are well developed algorithms to identify gene patterns from pairs of genomes, while discovering gene patterns from multiple genomes is a highly complex problem. Our paper proposes a pruning based algorithm to efficiently identify gene patterns in a large number of genome sequences, instead of using enumeration or a dynamic programming approach.

In this paper, we introduce a novel algorithm for mining gene patterns from genome sequences using interchangeable sets. We apply the proposed DISPattern algorithm on a set of prokaryotes genomes to discover gene patterns. Then we use these gene patterns to predict orthologous groups of certain genes.

## Methods

In this section, we present the definition of gene patterns and the algorithm to efficiently discover frequent gene patterns from genomes using interchangeable sets. The main difference between the gene pattern mining problem and the family pattern mining problem [10,11] is that the annotation of the entire set of genes is known in the family pattern problem while the annotation of some (or all) genes is unknown in the gene pattern mining problem. The family pattern mining problem can be considered as a special case of the gene mining problem. A genome can be represented *G *= {(*g*_1_, *s*_1_, *e*_1_), (*g*_2_, *s*_2_, *e*_2_),..., (*g*_*n*_, *s*_*n*_, *e*_*n*_)}, where *g*_*i *_are genes at the genome, and *s*_*i *_and *e*_*i *_are the starting position and ending position of *g*_*i*_. Two genes are considered **interchangeable **if they have the potential to exhibit same biological functions. There are several ways to determine whether two genes have the potential to exhibit same biological functions. One is to use known biological knowledge. If they belong to the same orthologous family (e.g., COG family), then they have the same biological function. In the case that this type of annotation information is unavailable, sequence comparison can be used for this purpose. If the pairwise sequence similarity of two genes is above a certain threshold, these two genes are considered as **interchangeable**. A more relaxed requirement may be applied for assessing whether two genes are interchangeable. For example, the triangle merge in [8] can be used for this purpose. If genes *g*_1_, *g*_2 _and *g*_3 _from three genomes are interchangeable, then they form a triangle. If genes *g*_1_, *g*_2 _and *g*_4 _form an other such triangle , then these two triangles can be merged. So genes *g*_1_, *g*_2_, *g*_3 _and *g*_4 _will be put into the same interchangeable gene sets.

Under this relaxation, triangle merges will be conducted until no triangle can be merged. The interchangeable gene sets are therefore maximal. This relaxation could give better flexibility for computing the interchangeable gene sets. On the other hand, one gene can be in several interchangeable gene sets, which enables the proposed algorithm to handle multi-domain genes. The formal definition of interchangeable gene sets and gene patterns is as follows:

**Definition 1 ***An ****interchangeable gene set****, or ****interchangeable set ****for short, is a non-empty set of interchangeable genes*.

**Definition 2 ***A ****gene pattern ****is a set of interchangeable gene sets and in the format GC *= [*F*_1_, *F*_2_,...,*F*_*m*_], *where F*_1_, *F*_2_,...,*F*_*m *_*are interchangeable gene sets*. *The ***length ***of a gene pattern is the number of interchangeable sets in the gene pattern. An unordered gene set *[*g*_1_, *g*_2_, ...,*g*_*m*_] *is called an ****unordered instance ****of GC if g*_*j *_∈ *F*_*j *_*for every j *(1 ≤ *j *≤ *m*). *A permutation on an unordered instance of GC is called an ****ordered instance ****of the gene patterns GC*.

For instance, a gene pattern *GC *= [{*g*_1_}, {*g*_2_, *g*_3_}, {*g*_4_}] has two unordered instances [*g*_1_, *g*_2_, *g*_4_] *and *[*g*_1_, *g*_3_, *g*_4_]. Moreover, both (*g*_1_, *g*_4_, *g*_2_) and (*g*_3_, *g*_1_, *g*_4_) are ordered instances of *GC*.

**Definition 3 ***A genome sequence GC *= {(*g*_1_, *s*_1_, *e*_1_), (*g*_2_, *s*_2_, *e*_2_),...,(*g*_*n*_, *s*_*n*_, *e*_*n*_)} ***supports ****a gene pattern GC *= [*F*_1_, *F*_2_,...,*F*_*m*_] *if and only if the reexist integers j*_1_, *j*_2_,...,*j*_*m*_, *such that*

• *A subsequence of G*, *G*_*sub *_= ($g_{j_{1}},g_{j_{2}},...,g_{j_{m}}$), *is an order edinstance of GC*.

• *For every j*_*i *_(1 ≤ *i *<*m*), $s_{j_{1+1}}-e_{j_{i}}\leq T_{gap}$, *where T*_*gap *_*is a pre-defined value called the ****gap threshold***.

*We call a gene pattern a ****frequent gene pattern ****if at least T*_*sup *_*genomes in a genome set support this pattern*. *T*_*sup *_*is called the support threshold*.

That is to say, if in a genome there exist *m *positions, where *m *is the length of a gene pattern, the genes at these positions in the sequence form an ordered instance of the gene pattern; and if in this genome the distance between any two consecutive genes of these *m *genes is no larger than the gap threshold *T*_*gap*_, *then *this genome is said to support the gene pattern.

**Example**: *GC *= [{*g*_1_}, {*g*_2_, *g*_3_}, {*g*_4_}] is a gene pattern, which contains three interchangeable sets: {*g*_1_}, {*g*_2_, *g*_3_} and {*g*_4_}. Let *T*_*gap *_be 300. Genome sequences {(*g*_4_, 200, 1000), (*g*_2 _1200, 2200), (*g*_1_, 2400, 3500)} and {(*g*_1_, 300, 1100), (*g*_3_, 1200, 2100), (*g*_4_, 2200, 3500)} (both support *GC *while genome sequence {(*g*_2_, 1000, 2900), (*g*_3_, 2300, 2900), (*g*_1_, 3000, 3500)} does not support *GC*. If the support threshold *T*_*sup *_is set to 3, and *GC *appears in 4 genomes, then *GC *is said to be a frequent gene pattern.

We invented a novel ***DISPattern ***algorithm to discover frequent gene patterns from a set of genomes. The DISPattern algorithm solves the following problem: Given a set of genome sequences, the interchangeable gene sets, the gap threshold *T*_*gap*_, and the support threshold *T*_*sup*_, we want to find frequent gene patterns among these genome sequences.

In theory, any combination of interchangeable sets can be a candidate for frequent gene patterns. If we enumerate all candidate patterns by combining any interchangeable sets, a set enumeration lattice [14] can be built. The top level nodes of the lattice are single interchangeable sets, and lower level nodes at the lattice are combinations of single interchangeable sets. We can then systematically search the entire set enumeration lattice for frequent gene patterns. When computing the support of a gene pattern, we compute the support of all ordered instances of *GC*. If the overall support is above the threshold, then *GC *is considered frequent.

However, in the set enumeration lattice, the number of candidates grows exponentially with the number of the interchangeable sets. Thus, the exhaustive search of the set enumeration lattice is not efficient or even not possible when the number of interchangeable sets is large. Therefore, a more efficient algorithm needs to be devised to solve the problem. In our proposed DISPattern algorithm, possible candidates for gene patterns are identified in two scans of the genome sequences, then are pruned by a reachability property. The DISPattern algorithm works as follows: first we record the information of the sequences in a data structure called reachable cases. Originally interchangeable sets in every reachable case could be a candidate for a frequent gene pattern, thus the number of candidate patterns is very large. Armed with the reachability property, we can first identify some pairs of interchangeable sets that can not be in a frequent gene pattern, then use these pairs to prune the set of reachable cases. After pruning the reachable cases, more pairs of interchangeable sets that cannot coexist in the same frequent gene pattern can be identified. This two-way pruning process is conducted iteratively. Finally the remaining reachable cases are verified to produce all frequent gene patterns.

**Figure 1:**
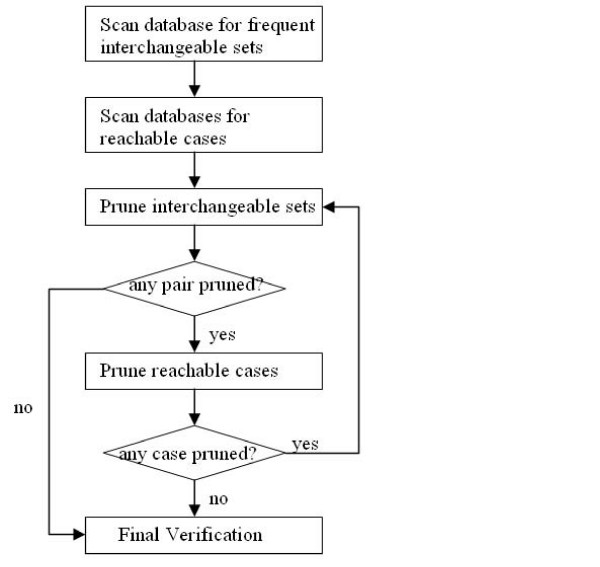
**DISPattern Algorithm**. This figure is the Flowchart of DISPattern algorithm.

The flowchart of the DISPattern algorithm is given in Figure 1. Three main phases of the DISPattern algorithm are explained in the remainder of this section.

### Initial Scanning

In the first phase of our algorithm, two scans of genome sequences are conducted. With the first scan, the frequency of every interchangeable set in all genomes is collected. If a gene belongs to multiple interchangeable sets, its occurrence counts for all of its interchangeable sets. Infrequent interchangeable sets, which can not participate in a frequent gene pattern, can be pruned after the first scan.

Next we define the terms of "reachable" and "reachable case" based on the given gap threshold *T*_*gap*_.

**Definition 4 ***In a genome sequence G *= {(*g*_1_, *s*_1_, *e*_1_), (*g*_2_, *s*_2_, *e*_2_),..., (*g*_*n*_, *s*_*n*_, *e*_*n*_)}, *genes g*_*i *_*and g*_*j *_(1 ≤ *i*, *j *≤ *n*) *are said to be reachable, if one of the following two cases happens*:

• *s*_*j *_- *e*_*i *_≤ *T*_*gap*_, *which means the gap between g*_*i *_*and g*_*j *_is no larger than *T*_*gap*_. *We say g*_*i *_and *g*_*j *_*are directly reachable.*

• *there exist integers m*_1_, *m*_2 _...*m*_*m *_(1 ≤ *i *<*m*_1 _<*m*_2 _... <*m*_*p *_<*j *≤ *n) such that *$s_{m_{1}}-e_{i}\leq T_{gap},s_{j}-e_{m_{p}}\leq T_{gap}$*and for every m*_*q*_(1 ≤ *q *<*p*), $s_{m_{q+1}}-e_{m_{q}}\leq T_{gap}$. *Genes *$g_{m_{1}},g_{m_{2}}...g_{m_{p}}$*are called the **intermediate set **of g*_*i *_and *g*_*j*_*. We also say g*_*i *_*and g*_*j *_*are reachable through *$g_{m_{1}},g_{m_{2}}...g_{m_{p}}$.

*Two interchangeable sets F*_1 _and *F*_2 _*are said to be reachable in a sequence if there exist g*_1_(*g*_1 _∈ *F*_2_), *g*_2 _(*g*_2 _∈ *F*_2_), *and g*_1_, *g*_2 _*are reachable in this sequence*.

A reachable case is therefore a case in which two interchangeable sets are reachable either directly or through some interchangeable sets in certain genome. The second scan of the genome sequences records all reachable cases in all genomes. These reachable cases are candidate gene patterns, which will be pruned in a later stage. Note that the initial scanning transforms an original gene sequence into a sequence of interchangeable sets. Some interchangeable sets may not be correct in terms of gene functions. Incorrect interchangeable sets are most likely pruned at the next stage ("Pruning Reachable Cases") since a gene pattern requires that multiple interchangeable sets appear in a set of genomes. The correctness of the final gene patterns prediction is empirically shown in the experimental section.

### Pruning Reachable Cases

At this phase, we prune the set of reachable cases. First we give the definition of "instance sequence", then we present a reachability property using the definitions of "reachable" and "instance sequence".

**Definition 5 ***If a sequence supports a frequent gene pattern, we call this sequence an ****instance sequence ****of the gene pattern*.

*In an instance sequence of a gene pattern, any two interchangeable sets of the pattern will be reachable either directly or through the interchangeable sets of this frequent gene pattern*.

A reachability property for frequent gene patterns can be identified as follows:

**Property 1 ***Any pair of interchangeable sets from a frequent gene pattern, say GC, have to be reachable in at least T*_*sup *_*number of sequences*. *Additionally*, *in every instance sequence of GC*, *any pair of interchangeable sets from GC are reachable using only other interchangeable sets from GC as the intermediate set*.

**Proof 1 ***The proof comes directly from the definitions of reachable and gene patterns. Assume two interchangeable sets F*_*i *_*and F*_*j *_*are in a frequent gene pattern, say GC*. *According to the definition of frequent gene patterns, GC occurs in at least T_*sup *_sequences. A gene pattern's occurrence in a sequence implies that in this sequence, F_*i*_, and F_*j *_are reachable either directly or through some interchangeable sets from GC. Therefore, in these T_*sup *_sequences*, *F*_*i*_, and *F*_*j *_*are reachable through interchangeable sets in GC only*.

Armed with the reachability property, we can prune the set of candidate patterns as follows. First, if we can identify pairs of interchangeable sets that are reachable in less than *T*_*sup *_sequences using any interchangeable set as intermediate sets, then these pairs of interchangeable sets can not be in any frequent gene pattern. This pruning is based on the first portion of the reachability property. Moreover, we can use these pruned pairs to further prune the remaining pairs of interchangeable sets. The following lemma is derived to support this pruning step.

**Lemma 1 ***If in one sequence, two interchangeable sets from a frequent gene pattern, e.g. GC, are only reachable through a certain interchangeable set which does not belong to GC, then this sequence cannot be an instance sequence of the gene pattern GC*.

The above lemma, which is easy to derive from the reachability property, can be used to prune the number of instance sequences of a candidate pattern. The intuition behind this pruning is: In order to confirm two interchangeable sets *F*_1_, *F*_2 _are in an frequent gene pattern *GC*, we need to identify at least *T*_*sup *_instance sequences of *GC*. If we know another interchangeable set *F*_3 _cannot be in a frequent gene pattern with *F*_1_, and in some sequence *F*_1 _is only reachable to *F*_2 _through *F*_3_, then this sequence cannot be an instance sequence of *GC *according to the above lemma.

After the above pruning, two interchangeable sets may become reachable in fewer sequences. This may help us to further identify pairs of interchangeable sets that cannot form a frequent gene pattern. After identifying these pairs, we conduct the pruning of the reachable cases again since more pairs are known to be unable to form a frequent gene pattern. This two-way pruning is iteratively conducted until no interchangeable set pairs can be pruned or no reachable cases can be pruned, as illustrated in the flowchart of the algorithm (Figure 1).

### Final Verification

In the last verification step, for each remaining reachable case, we sort the labels of interchangeable sets by their lexical order. By sorting interchangeable set labels, we can transform different permutations of a gene pattern into a unique representation. For example, the reachable case that interchangeable set *F*_1 _is reachable to *F*_2 _through *F*_3 _and the case that *F*_3 _is reachable to *F*_2 _through *F*_1 _are both transformed to the same list *F*_1_*F*_2_*F*_3_. Now we count the number of sequences that each list occurs in by traversing all reachable cases. If a list of interchangeable sets occurs in *T*_*sup *_sequences, then these interchangeable sets form a frequent gene pattern.

## Results and Discussion

In this section, we present the experimental results of our proposed gene pattern mining model. Compared with previous gene cluster discovery methods, e.g., [10,11], the gene pattern model proposed in this paper can handle the genes without annotation. In [10,11], it is required that the annotation of every gene is known and each gene belongs to one family. However, in the problem context studied in this paper, the annotations of some genes are not available. Thus, our gene pattern model can be used to (1) predict the annotation of a gene and (2) find gene clusters with non-annotated genes. In this section, we will analyze the effectiveness of the gene pattern model and methods with respect to these two goals.

### Gene Patterns from Four New Genomes

We also performed other experiments using four genomes, *Azotobacter vinelandii*, *Bdellovibrio bacteriovorus*, *Myxococcus xanthus*, and *Rhodospirillum centenum*. These four genomes are good for testing our algorithm since they belong to Proteobacteria and they are not well characterized, that is, family information is not available. One genome, *Rhodospirillum centenum*, is a new genome that has not been published but obtained from our collaboration at Indiana University. Since COG assignments are not available for these four new genomes, we used best hits and triangle merging method to construct the interchangeable gene sets in this case. As shown at the website [15], our algorithm successfully predicted many biologically meaningful gene patterns according to the current annotations of the genomes. Predicted gene patterns can be used either to confirm functions of genes that were predicted by similarity match or to predict de novo functions, especially for genes with annotation of hypothetical or unknown functions. For example, one gene pattern covers three cell division proteins FtsZ, FtsA and FtsQ among three genomes. Another gene pattern contains electron transfer flavoprotein subunits which appear in *Bdellovibrio bacteriovorus*,*Myxococcus xanthus*, and *Rhodospirillum centenum*. Clustering non-annotated genes can only be done in the gene patterns proposed in this paper, but not by the family pattern methods proposed in [10,11].

### Application of Gene Patterns to Ortholog Prediction

To further show the effectiveness of our gene pattern model, we used the discovered gene patterns to predict orthologous groups of genes.

Ortholog detection is critically important for accurate functional annotation, and has been widely used to facilitate studies on comparative and evolutionary genomics. In [16], the authors proposed an approach for identifying orthologous gene pairs between mouse and human genomes. The approach combines the mutual selection of the best BLAST hits between human and mouse transcripts, and inferring gene orthologous relationships based on sharing synthetic anchors, collocating in the same synthetic blocks and sharing the same annotated protein function. However, their approach can only find pairs of orthologs, not groups of orthologs. Also, their method is restricted to a the comparison between mouse and human genomes, which may not be widely applicable to other species.

Our method of ortholog discovery consists of three steps. First, pair-wise protein sequence comparisons are conducted to assign corresponding genes whose family information are unknown to some candidate orthologous groups. Then using these candidate orthologous groups as interchangeable sets, we discover gene patterns from genome sequences by the DISPattern algorithm. Finally the discovered patterns are used to predict the orthologous groups of genes. These three phases are explained in details as follows: In the first step, the BLAST program [17] is used to perform pair-wise protein sequence comparison. The unassigned genes, whose gene family information is unknown, are classified into some candidate orthologous groups according to the protein sequence similarity. Two types of orthologous groups are used as candidate groups for an unassigned gene. First, the orthologous groups of the unassigned gene's best hit on any genome sequence will be used. Second, if the unassigned gene is the best hit of a gene of another genome, that gene's orthologous groups will also be used. In this way, an unassigned gene is classified to multiple existing orthologous groups. We choose the top-K most common groups as the candidate groups of this gene, where K is usually set to a small number (3 to 5). If K is set to 1, then each gene will be assigned to the annotation to which the gene is most similar. However, in many cases, the gene should not be assigned to the most similar annotation. These candidate orthologous groups will be further pruned by the gene patterns discovered in the next step.

After assigning each gene into its candidate orthologous groups, we use these candidate orthologous groups as interchangeable sets in the DISPattern algorithm. We run DISPattern to discover frequent gene patterns from these genomes. For each interchangeable set in the discovered gene patterns, we also record which gene appears in each genome for the interchangeable set.

Once the gene patterns are discovered from genome sequences, we analyze these gene patterns to assign genes to their orthologous groups. If a gene appears in a gene pattern as a member of an orthologous group, then this gene is assigned to this orthologous group. Other candidate orthologous groups assigned to this gene previously will be pruned. If one gene appears in multiple gene patterns as different orthologous groups, we use the following rules to solve conflicts:

• If multiple patterns cover the same gene, the longest pattern will be used for prediction

• If all patterns are of the same size, the pattern with the highest support will be used for prediction

We discovered that in many cases, the gene will be assigned to the annotation to which it is not most similar. If a gene never participates in any gene patterns (This is possible since the gene patterns will not cover every gene in the genomes), then we use bi-directional best hits(BBH) to assign this gene to an orthologous group. In BBH, to predict the orthologous group of a gene, we count the COG assignments of this gene's bi-directional best hits (BBHs) in other genomes. The most common COG assignment of its BBHs is considered as the predicted orthologous group.

As BBH only uses the best bi-directional hits as the ortholog predictions, it misses a significant number of orthologs. By introducing the gene patterns, we allow more ortholog candidates at the first stage for each gene to be predicted. The gene patterns help to decide which ortholog candidate is more likely to be the correct prediction. The role of gene patterns is important since it filters out incorrect predictions using the intrinsic biological meaning of these patterns. As the experimental results show, the gene patterns will correct a large portion of mistakes made by BBH method.

In this section, we present the experimental results of our proposed computational ortholog discovery approach based on gene patterns. To validate the effectiveness of this approach, the Cluster of Orthologous Groups (COGs) were used as the benchmark. COG [8] uses single-direction best hits(Bet) and triangle merging to construct orthologous groups. We compared our results against those of Bi-directional Best Hits (BBH), which is another computational method for ortholog prediction and it is used at the first stage of COG. In the experiments, we removed the COG information of certain genes, then used our method and BBH to predict these genes into orthologous groups. We evaluated the results using the original COG assignments in terms of recall and precision. The recall is defined as *C*_*correct*_/*C*_*removed*_, where *C*_*correct *_is the number of genes correctly assigned by our method, and *C*_*removed *_is the total number of genes whose COG information is removed. The precision is defined as *C*_*correct*_/*C*_*assigned*_, where *C*_*assigned *_is the total number of ortholog assignment our method predicts. In essence, the recall measures how likely that a gene can be correctly annotated while the precision measures how likely an assignment is correctly. In our method, since we use the above rules to solve conflicts, one gene is predicted to exactly one orthologous group, therefore the recall of our method is the same as the precision.

Two types of experiments were conducted to evaluate our proposed method. The first scenario was to remove the COG information of randomly chosen genes from multiple genomes, then try to recover their orthologous groups. The second scenario was to remove the COG assignments of all genes in a single genome, then try to predict the orthologous groups of every gene.

In both scenarios, we downloaded ten genomes from National Center for Biotechnology Information (NCBI) web site. Each genome contains thousands of genes along with their positions at the genome. These genes are pair-wise compared by the BLAST program. The full list of these 10 sequences is available at our website. For the DISPattern algorithm of gene pattern discovery, the distance threshold was set to 300 base pairs and the support threshold was set to 3. The execution time of gene pattern generation from the 10 genomes (after interchangeable sets are constructed) is around 5 seconds. We also experimented with the gene pattern mining method on a much large data set. For example, the execution time for discovering gene patterns from 120 genomes from the NCBI website is between 40 to 80 seconds depending on the support thresholds.

Next we will present the experimental results in both scenarios.

#### Predicting Genes on Multiple Genomes

In this setting, we randomly removed the COGs of 2000 genes from any genome. BBH resulted in an average recall of 87%, while our model achieved a recall of 91.2%. As stated above, our approach also had a precision of 91.2%, while BBH's precision was 87%.

#### Predicting Genes on Single Genome

In this setting, we removed the COGs of all genes on one genome (called the target genome), then kept the COG information of other genomes as reference genomes. The approach proposed above was then used to predict the orthologous groups of genes on the target genomes. We conducted the experiments on different target genomes and computed the average recall and precision of both BBH and our method. On average our method achieved a recall/precision of 90.1% while BBH had a recall and precision of 87.3%. The average improvement on recall and precision of our method was thus 3%.

### Parameters

In this subsection, we examine how the parameter settings affect the results of ortholog prediction. Two parameters can be varied in the ortholog discovery approach proposed above: the support threshold for frequent gene patterns *T*_*sup*_, and the top-*K *value when choosing the candidate orthologous groups for a gene.

**Figure 2:**
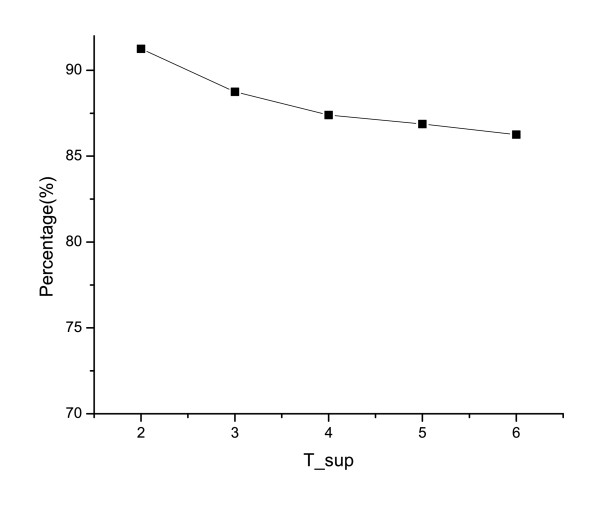
**Accuracy of DISPattern w.r.t. support**. Different support thresholds yield different sets of DISPattern. By applying these different sets of patterns, different gene annotations may be obtained. This figure shows the recall and precision of the gene annotation w.r.t. the support threshold. Since each gene is assigned to one group, the precision is the same as the recall.

In the gene pattern mining algorithm, *T*_*sup *_is the support threshold which defines frequent patterns. Varying the support threshold will vary the number of patterns discovered. The lower the *T*_*sup *_value is, the more patterns will be discovered. The recall and precision of our proposed ortholog discovery approach change with different support thresholds *T*_*sup*_, which is plotted in figure 2. In this test, a gene is mapped to one ortholog group, thus the recall is equal to the precision.

**Figure 3:**
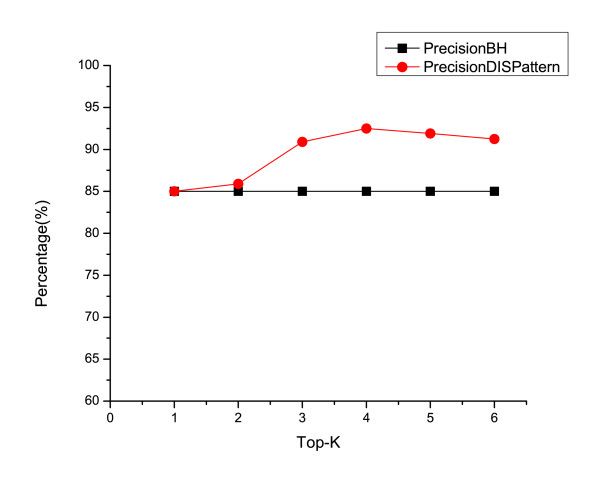
**Accuracy of DISPattern w.r.t. Top-k Ortholog Candidates**. When assigning a gene, we use the *k *most common Orthologs as candidates. This figure shows the accuracy when a different number of ortholog candidates are used.

To assign a gene to its candidate orthologous groups, we choose the top-*K *most common orthologous groups of this gene's hits on other genomes. When *K *is set to 1, it is essentially the same as BBH. When *K *is larger, more candidate groups will be assigned to each gene. The effect of the *K *value on the precision and recall of the ortholog discovery method is shown in figure 3. The recall/precision increases when *K *changes from 1 to 4. However, the recall and precision will decrease slightly when *K *becomes larger than 4. The reason for this is that assigning each gene to too many candidate orthologous groups introduces too much noise. Even when the pruning step is conducted later by applying the discovered patterns, the errors introduced by the noise will not be fully removed. In this experiment, the BHH is used as the baseline model. Since the BHH always assigns a gene to the COG group with best hits, the recall of BHH stays constant. (BHH also has the same recall and precision since it assigns a gene to only one COG.) The benefit of our method comes from the fact that the gene pattern can be used as a tool in finding the correct orthologous group if a gene can be grouped into multiple groups, i.e., the gene has high similar with genes in multiple groups. When *K *= 1, each gene is assigned to one orthologous group. Thus, there is no benefit of our method. However, with a larger *K*, i.e., a gene may be associated with multiple groups, our method can prune out the incorrect group assignment. For instance, if gene *g *has higher similarity with genes in group *A *than genes in group *B*, the BBH will assign *A *to *g*. However, *g*'s true annotation may be *B*. In this case, the gene pattern may be able to correctly assign *g *to *B *based on the location of gene *g*.

### Comparison on Example Genomes

Here we present the detailed comparison of our method and BBH on a few example target genomes. The first genome to test is the complete genome of *Mycobacterium tuberculosis H37Rv *(NC_000962). The second genome is *Bordetella pertussis Tohama I *(NC_002929). The summary of the results is presented in Table 1. In the complete genome of *Mycobacterium tuberculosis H37Rv *there are 3927 genes in total, in which 2756 genes have COG assignments. BBH correctly predicted 2394 genes out of 2756, which resulted in a recall of 86.8%. Our approach outperformed BBH by successfully predicting 2576 genes which leads to a recall of 93.5%. 186 genes were correctly predicted by our method but not by BBH. Table 2 are some example genes correctly predicted by our method but not predicted by BBH.

**Table 1:** Results on two example target genomes

Genome	NC_000962	NC_002929
No. of genes	2756	2723
Recall of BBH	86.8%	89.4%
Recall of DISPattern	93.5%	91.0%
Precision of BBH	86.8%	89.4%
Precision of DISPattern	93.5%	91.0%
No. of errors by BBH	186	106
No. of errors by DISPattern	66	61

**Table 2:** Example genes correctly predicted by DISPattern but not predicted by BBH

Gene(id)	COG	Pattern	Occurrence
Rv2141c (id: 57116951)	COG0624 Acetylornithine deacetylase Succinyl-diaminopimelate desuccinylase and related deacylases	{COG0167 COG0624 COG1881}	NC_002755 NC_002945 NC_000962
Rv0558 (id: 57116754)	COG2226 Methylase involved in ubiquinone/menaquinone biosynthesis	{COG0438 COG2226}	NC_002935 NC_004369 NC_002944 NC_002755 NC_002945 NC_000962
Rv1319c (id: 15608459)	COG2114 Adenylate cyclase, family 3 (some proteins contain HAMP domain)	{COG1637 COG2114 COG2114 COG2114}	NC_002755 NC_002945 NC_000962
Rv1034c (id: 15608174)	COG3039 Transposase and inactivated derivatives, IS5 family	{COG0642 COG0745 COG2156 COG2216 COG3039}	NC_002755 NC_002945 NC_000962
nrp (id: 15607243)	COG3320 Putative dehydrogenase domain of multifunctional non-ribosomal peptide synthetases and related enzymes	{COG0227 COG0474 COG0523 COG0664 COG2217 COG3320 COG3336}	NC_002755 NC_002945 NC_000962

In the complete genome of *Bordetella pertussis Tohama I*, there are 3436 genes in total, in which 2723 genes have COG assignments. BBH had a recall of 89.4% (2436 correctly predicted genes out of 2723), while our approach predicted 2492 correctly out of 2723 genes, which has a recall of 91.5%.

Table 3 are some example genes correctly predicted by our method but not predicted by BBH.

**Table 3:** Example genes correctly predicted by DISPattern but not predicted by BBH

Gene(id)	COG	Pattern	Occurrence
BP0202 (id: 33591446), BP0203 (id: 33591447), BP0210 (id: 33591454), BP0211 (id: 33591455)	COG2801 Transposase and inactivated derivatives	{COG2801 COG2801}	NC_005090 NC_002662 NC_004369 NC_002929 NC_003295 NC_003902 NC_004741 NC_004337
BP0153 (id: 33591402)	COG3565 Predicted dioxygenase of extradiol dioxygenase family	{COG0111 COG0583 COG0642 COG3019 COG3565}	NC_002929 NC_002928 NC_002927
BP0778 (id: 33591402)	COG0318 Acyl-CoA synthetases (AMP-forming)/AMP-acid ligases II	{COG0318 COG0604 COG1802 COG4625}	NC_002929 NC_002928 NC_002927

## Conclusion

In this paper, we proposed a novel algorithm for mining gene patterns in multiple prokaryote genomes using interchangeable sets for the cases where family classification information is not available. Unlike the existing algorithms that predict gene patterns in a pair of genomes using the dynamic programming technique, our algorithm is highly scalable as shown in the experiments with genomes of 97 species. The scalability of our algorithm is achieved by extending the pattern mining technique from the data mining community. We also showed that our pattern mining algorithm can be used for detecting orthology correctly as gene patterns can be used as *context *of gene matching. In an experiment detecting orthologies in 10 genomes, our algorithm achieved an average of 3 % increase in recall compared to BBH without sacrificing the precision of ortholog detection.

## Authors' contributions

MH did the implementation and experiment of gene pattern algorithm. KC and SK helped prepare the biological data and analyze the biological meaning of the discovered patterns. WS finalized the paper. JY supervised the entire project. All authors have read and approved the final version of the manuscript.
